# Supplementary material for: Concurrent prediction of RNA secondary structures with pseudoknots and local 3D motifs in an integer programming framework
Source: Bioinformatics. 2024 Jan 16;40(2):btae022. doi: 10.1093/bioinformatics/btae022 (PMC10868335; doi:10.1093/bioinformatics/btae022)
Supplement: btae022_Supplementary_Data [file btae022_supplementary_data.pdf]

Concurrent prediction of RNA secondary structures with pseudoknots and local 3D motifs in an Integer Programming framework

Gabriel Loyer, Vladimir Reinharz

December 1, 2023

1 Supplementary material

1.1 Full Integer Programming Model

The hairpins insertion that are composed of only one strand are constrained by Eq. 1, which was modified to only consider base pairs in level 1. The insertion of interior loops and bulges must first ensure that strands are placed in acceptable positions (Eq. 2) and that the motif must fill at least 2 unpaired positions, ensuring information is added to the system (Eq. 3).

$$\forall_{(x,k,l)\in Seq_1^1}:$$
$$C_{k,l}^{x,1} \leq \sum_{\substack{(u,v)\in\mathcal{B}\\ k-1\leq u\leq k\wedge\\ l\leq v\leq l+1}} (D_{u,v}^1) + \sum_{\substack{(\tilde{x},\tilde{k},\tilde{l})\in Seq_1^2\\ \tilde{l}=k-1}} C_{\tilde{k},\tilde{l}}^{\tilde{x},1} + \sum_{\substack{(\tilde{x},\tilde{k},\tilde{l})\in Seq_2^2\\ \tilde{k}=l+1}} C_{\tilde{k},\tilde{l}}^{\tilde{x},2} \quad (1)$$

$$\forall(u,v)\in\mathcal{B},\ \forall x\in Mot^2:$$
$$-n(1-D_{u,v}^1)\leq$$
$$\sum_{\substack{(x,k,l)\in Seq_1^2\\ l<u\vee v<k}} C_{k,l}^{x,1} - \sum_{\substack{(x,k,l)\in Seq_2^2\\ l<u\vee v<k}} C_{k,l}^{x,2} \leq n(1-D_{u,v}^1) \quad (2)$$

$$\forall(x,k,l)\in Seq_1^2, \forall(x,\tilde{k},\tilde{l})\ |$$
$$\tilde{k}>l\wedge 2\cdot\sum_{\substack{(u,v)\in\mathcal{B}\\ k\leq u\leq l\wedge\tilde{k}\leq v\leq\tilde{l}}} 1 + \sum_{\substack{(u,v)\in\mathcal{B}\\ k\leq u\leq l\oplus\tilde{k}\leq v\leq\tilde{l}}} 1 \geq l-k+\tilde{l}-\tilde{k}+1\in Seq_2^2:$$
$$C_{k,l}^{x,1}+C_{\tilde{k},\tilde{l}}^{x,2}\leq 1 \quad (3)$$

The k-way junctions admissibility of insertion is decided in Eq. 4, ensuring that each strand can be reached without crossing the base pairs in the first level. This is equivalent to Eq. 2 for the interior loops.

$$\forall j \geq 3,\ \forall (u,v) \in \mathcal{B}:\ -n(1 - D^1_{u,v}) \leq$$
$$(j-1) \cdot \sum_{\substack{(x,k,l) \in Seq^j_1 \\ u \leq k \leq l \leq v}} C^{x,1}_{k,l} - \sum_{\substack{1 < i \leq j \\ (x,k,l) \in Seq^j_i \\ u \leq k \leq l \leq v}} C^{x,i}_{k,l} \leq n(1 - D^1_{u,v}) \tag{4}$$

An important feature of RNA structure is that their sequence is ordered, from the $5'$ to the $3'$ end, and that it is not symmetric. In a motif, an order is defined over the strands following that direction. The model constrains where a strand in a motif can be placed given the insertion of the previous (Eq. 5) or next (Eq. 6) strand of the same motif. An important consideration is that at the end there must exist a mutually exclusive decomposition of the strands such that each inserted motif is complete, even if many copies are found (Eq. 7).

$$\forall\ 1 < i \leq j,\ \forall (x,k,l) \in Seq^j_i:\ C^{x,i}_{k,l} \leq \sum_{\substack{(x,\tilde{k},\tilde{l}) \in Seq^j_{i-1} \\ \tilde{l} < k-5}} C^{x,i-1}_{\tilde{k},\tilde{l}} \tag{5}$$

$$\forall\ 1 \leq i < j,\ \forall (x,k,l) \in Seq^j_i:\ C^{x,i}_{k,l} \leq \sum_{\substack{(x,\tilde{k},\tilde{l}) \in Seq^j_{i+1} \\ l+5 < \tilde{k}}} C^{x,i+1}_{\tilde{k},\tilde{l}} \tag{6}$$

$$\forall\ j > 1,\ \forall x \in Mot^j,\ \forall 1 < i \leq j:$$
$$\sum_{(x,k,l) \in Seq^j_1} C^{x,1}_{k,l} - \sum_{(x,\tilde{k},\tilde{l}) \in Seq^j_i} C^{x,i}_{\tilde{k},\tilde{l}} = 0 \tag{7}$$

1.2 Predicting canonical interactions in motifs

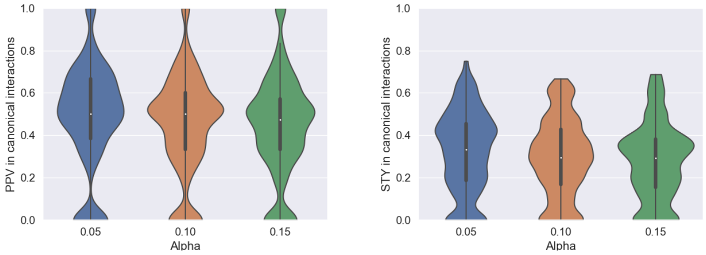

(a) PPV (b) STY

Figure S1: **Predicting canonical and Wobble interactions in motifs**. For α values of $0.05, 0.1, 0.15$ that more than half of the canonical and Wobble base pairs in the motifs are correctly predicted, and 40% of them are generally captured.

1.3 Predicting non-canonical interactions in motifs

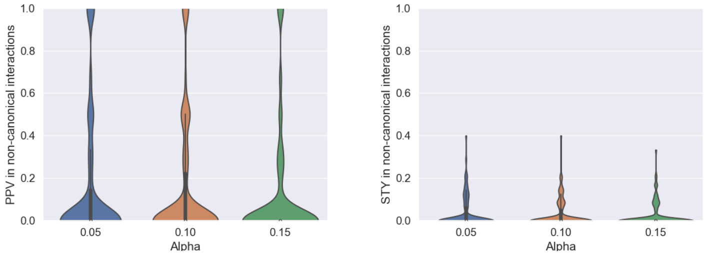

(a) PPV (b) STY

Figure S2: **Prediction accuracy of non-canonical base pairs in motifs**. True positives are the non-canonical base pairs at positions where one motif is inserted in the sequence. They are composing at most 15% of the interactions in the inserted motifs, and are hard to predict.

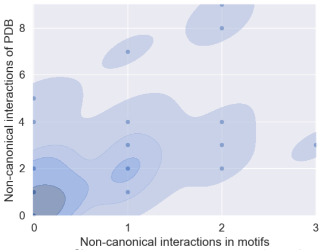

Figure S3: **Non-canonical interactions** distribution of the number of non-canonical interactions that are observed at motifs inserted locations. On the y-axis the number in the real structure, on the x-axis how many are annotated in the inserted motif.

1.4 RNAMoIP on alignments

Due to the nature of the sequence alignment, we relax the procedure to insert motifs as follows. RNAMoIP predicts the structure of 1 sequence that can be enhanced with an alignment. All columns that are gaps in the sequence of interest are discarded. Therefore, for a motif component $C^{x,1}_{k,l}$ the position k, l are the same in the structure for which we are doing the prediction, and the alignment.

We first identify for each sequence, without gaps, positions where each motifs can fit. For each of these, we count in the alignment the fraction of other sequences that are at most at a Hamming distance of 1. If that ratio is above 50% we consider that the motif can be inserted at these positions. Formally, we define a function

$$\text{found\_in}(C^{x,1}_{k,l}) \to [0, 1]$$

such that: $\text{found\_in}(C^{x,1}_{k,l})$ returns the fraction of subsequences in the alignment between positions k, l that are at most at Hamming distance one from the motif $C^{x,1}$. Then we can have the normalizing function:

$$\text{sim}(C^{x,1}_{k,l}) = \begin{cases} 1 & \text{if the motif matches exactly the sequence in k,l} \\ 0 & if \text{found\_in}(C^{x,1}_{k,l}) < 0.5 \\ \text{found\_in}(C^{x,1}_{k,l}) & else \end{cases}$$

.

Finally the updated objective function when an alignment is provided becomes:

$$\max \quad \alpha \sum_{x \in Mot^j} \left((|x|)^{\,2} \times \sum_{(x,k,l) \in Seq^j_1} C^{x,1}_{k,l} \ \text{sim}(C^{x,1}_{k,l}) \right) + 10(1-\alpha) \times \sum_{(u,v) \in \mathcal{B}} \sum_{q=1}^{m} D^q_{u,v} \ p(u,v) \ \beta^q \qquad (8)$$

Weight of motif due to alignment

Motif inserted at position (k, l)

Motif length

Base pair (u, v)

Probability of the base pair (u, v)

Weight of level q

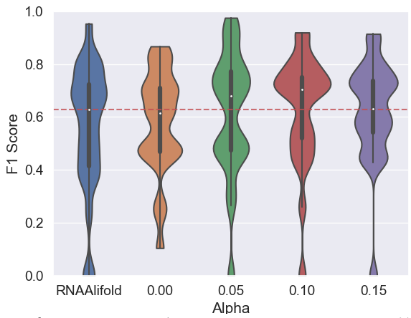

Figure S4: **Alignment-free secondary structure prediction accuracy**. Result of the alignment's dataset without using the alignment informations.

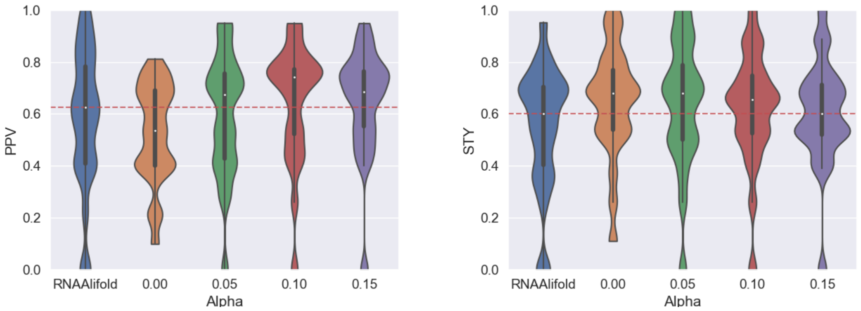

(a) PPV

(b) STY

Figure S5: **Alignment-free secondary structure prediction accuracy**. Result of the alignment's dataset without using the alignment informations.

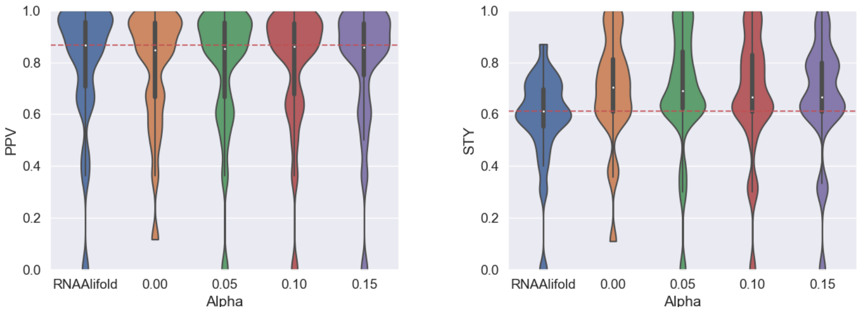

(a) PPV

(b) STY

Figure S6: **Alignment-based secondary structure prediction accuracy**. Result of the alignment's dataset with the help of the alignment informations.

1.5 Complete tool analysis

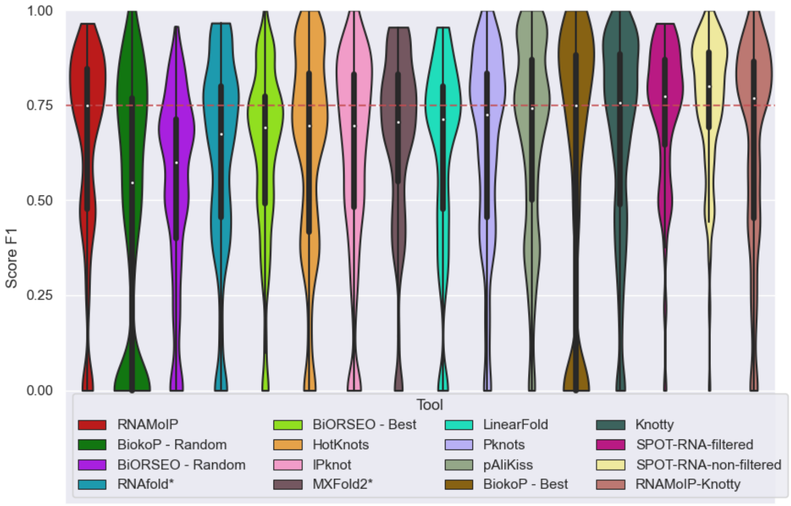

Figure S7: **Tools comparison of F1 scores**. Include two versions of SPOT-RNA. As in Fig 6 SPOT-RNA is evaluated on the subset of sequences not in its training set. We also show the results on the entire dataset, highlight the overfitting if not careful in the separation of test and train set.

1.6 Computation time benchmark

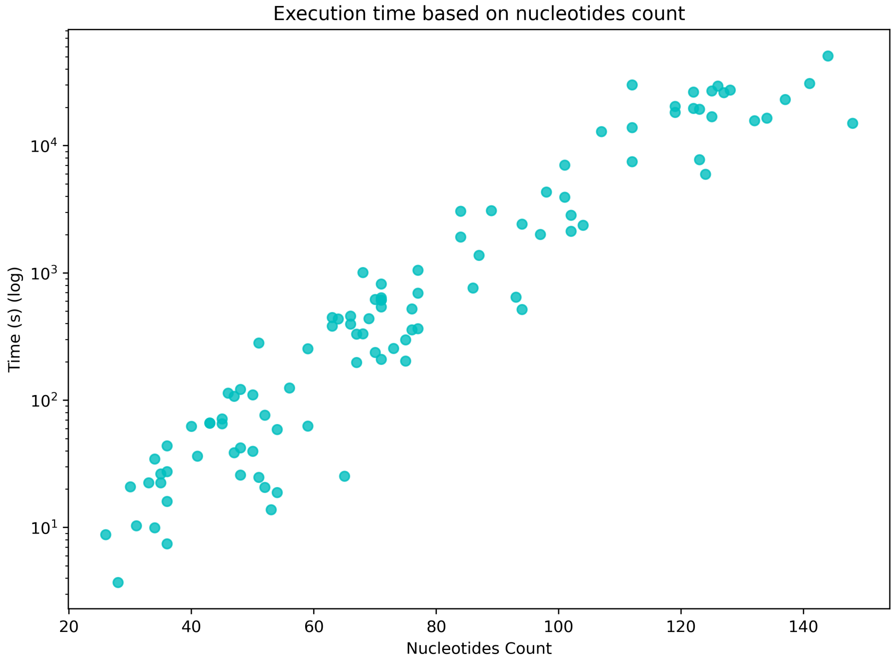

Figure S8: **Execution Time-based on nucleotide count of the sequence at** $\alpha = 0.1$. A maximum of 10^4s was allowed, and 14 sequences didn't return a solution in that time.
